# An Epidemiological Reappraisal of the Familial Aggregation of Prostate Cancer: A Meta-Analysis

**DOI:** 10.1371/journal.pone.0027130

**Published:** 2011-10-31

**Authors:** Michał Kiciński, Jaco Vangronsveld, Tim S. Nawrot

**Affiliations:** 1 Centre for Environmental Sciences, Hasselt University, Diepenbeek, Belgium; 2 Centre for Environmental Sciences, Hasselt University, Diepenbeek, Belgium; 3 Department of Public Health, Leuven University (KULeuven), Leuven, Belgium; University of Ottawa, Canada

## Abstract

Studies on familial aggregation of cancer may suggest an overall contribution of inherited genes or a shared environment in the development of malignant disease. We performed a meta-analysis on familial clustering of prostate cancer. Out of 74 studies reporting data on familial aggregation of prostate cancer in unselected populations retrieved by a Pubmed search and browsing references, 33 independent studies meeting the inclusion criteria were used in the analysis performed with the random effects model. The pooled rate ratio (RR) for first-degree family history, i.e. affected father or brother, is 2.48 (95% confidence interval: 2.25–2.74). The incidence rate for men who have a brother who got prostate cancer increases 3.14 times (CI:2.37–4.15), and for those with affected father 2.35 times (CI:2.02–2.72). The pooled estimate of RR for two or more affected first-degree family members relative to no history in father and in brother is 4.39 (CI:2.61–7.39). First-degree family history appears to increase the incidence rate of prostate cancer more in men under 65 (RR:2.87, CI:2.21–3.74), than in men aged 65 and older (RR:1.92, CI:1.49–2.47), p for interaction = 0.002. The attributable fraction among those having an affected first-degree relative equals to 59.7% (CI:55.6–63.5%) for men at all ages, 65.2% (CI:57.7–71.4%) for men younger than 65 and 47.9% (CI:37.1–56.8%) for men aged 65 or older. For those with a family history in 2 or more first-degree family members 77.2% (CI:65.4–85.0%) of prostate cancer incidence can be attributed to the familial clustering. Our combined estimates show strong familial clustering and a significant effect-modification by age meaning that familial aggregation was associated with earlier disease onset (before age 65).

## Introduction

Prostate cancer is one of the most common cancers among males in developed countries [1]. A lot of evidence shows that a family history of the disease is an important risk factor [1], [2]. In 2003, three meta-analyses evaluated the increase in the risk of prostate cancer in relatives of affected men [3]–[5]. Since then, familial clustering has been assessed in a number of new populations. Furthermore, more recent data is available from the big cohorts in Sweden [6] and the US [7]. We studied all the data available up to September 2010 to assess the strength of prostate cancer familial aggregation. In order to evaluate the impact of a family history of prostate cancer on the disease incidence, we also estimated the attributable fractions among men with affected relatives.

## Methods

### Searching

We browsed the PubMed database using the search term ‘(prostate cancer) and (family history)’. The last update was performed September 21, 2010. Out of 801 initially identified articles, 53 reports provided data on the relationship between family history and risk of prostate cancer in an unselected population of men (see Figure S1). Case-control studies using selected populations as cases (example: patients undergoing prostatectomy [8], [9]) and cohort studies with a specific cohort (example: smokers [10]) were excluded to avoid bias or heterogeneity due to these population characteristics. Additional 21 study reports were found through the references of the studies identified via the PubMed database.

### Selection

74 relevant articles were coded. As quality control, we considered study design, control for age and the way family history was ascertained. Cohort studies and case-control studies reporting age-adjusted estimates or using age-matched controls were included. Cross-sectional studies [11]–[17] were excluded. Two studies in which the attempt to match for age did not result in a similar age of the cases and the controls [18], [19] and one study in which the controls were not age-matched and age-adjusted estimates were not reported [20] were excluded. Additionally, one case-control study was excluded [21], because none of the participants reported a family history of prostate cancer. The study of McCahy et al. [22] was not used in the main analysis, because it was characterized by clearly outlying results (odds ratio for first-degree family history 17.83). The influence of exclusion of this study on the estimates was assessed in the sensitivity analysis.

Five studies [23]–[27] were excluded, because the investigated type of familial clustering did not correspond to any of the exposures and reference categories considered in this meta-analysis (affected first-degree relatives, i.e. father and/or brother(s), versus not, affected father versus not, affected father versus no affected first-degree relatives, affected brother(s) versus not, affected brother(s) versus no affected first degree-relatives, affected first or second-degree relative(s) versus not, two or more affected first-degree relatives versus no history in first-degree relatives). Two articles [28], [29] were excluded because of the lack of definition of ‘family history’.

Duplications in study populations were avoided so that each pooled estimate was based on independent studies. In case of an overlap between populations from several studies using the same design, a case-control study with the largest number of participants or the most recent cohort study was included. The case-control study reported by Negri et al. [30] was preferred over Gallus et al. [31] and Randi et al. [32], and Krain, 1974 [33] over Krain, 1973 [34]. Out of many reports based on the Swedish cancer register [6], [35]–[45], only the most recent [6] was used. Similarly, the most recent findings were included from the studies using the Utah population database [7], [46], [47] and the US Health Professionals Cohort [48], [49]. When there was a population overlap between studies using a different design, the cohort study [6] was preferred over the case-control studies [50]–[53], and the nested case-control study [7] over the study of West et al. [54]. A summary of the 25 case-control studies [30], [33], [55]–[77] and 8 cohort-based studies [6], [7], [49], [78]–[82] included in the analysis is presented in Table 1 and Table 2.

**Table 1:** Summary of the case-control studies included in the analysis.

First author and reference	Date	Place	Race	Mean age at diagnosis	No. cases	No. controls	Controls^A^
Beebe-Dimmer^55^	1996–2002	USA: Michigan	African	65	121	179	P C
Fincham^56^	1981–1983	Canada: Alberta	Caucasian	NA, age 45 or older	382	625	P C M
Ghadirian^57^	1989–1993	Canada: Montreal, Toronto, Vancouver	Caucasian	NA, median 70	640	639	P D M
Glover^58^	1998 B	Jamaica: Kingston	African	73,3	263	263	H C M
Hayes^59^	1986–1989	USA: Atlanta, Detroit, New Jersey	Mixed	61,5^C^	905	1264	P C M
Honda^60^	1979–1982	USA: Los Angeles County	Caucasian	NA, 60 or younger	216	216	P C M
Justine^61^	2001–2002	Australia: Perth	Caucasian	63,8 C	560	450	P C M
Kolonel^62^	1977–1983	USA: Hawaii	Mixed	NA	452	899	P C M
Krain^33^	1971–1972	USA: Los Angeles	Mixed	median 69	210	215	H C M
Lesko^63^	1992–1994	USA: Massachusetts	Caucasian	NA, median 65, age 70 or less	563	703	P D M
Magura^64^	2004–2006	USA: North Dakota	Caucasian	64,2 C	312	319	H C M
Mettlin^65^	1995 B	USA: Buffalo	Caucasian	67,6	1271	1909	H C M
Negri^30^	1991–2002	Italy	Italian	65,7 C	1294	1451	H C
Rovito^66^	1998–2001	USA: New York	Caucasian	63,3 C	152	161	H C M
Rybicki^67^	2001–2004	USA: Detroit	Mixed	NA, median 63	637	244	P C M
Salinas^68^	1993–1996, 2002–2005	USA: King County, Washington	Caucasian	59,9	1211	1208	P C M
Schuman^69^	1977 B	USA: Minnesota	Caucasian	NA, median 64	36	41	H D M
Spitz^70^	1985–1989	USA: Texas	Caucasian	66,2	378	383	H C M
Staples^71^	1994–1998	Australia: Melbourne, Sydney, Perth	Caucasian	60	1475	1405	P D M
Steele^72^	1968–1969	Canada: Ontario	Caucasian	69	39	39	H C M
Stone^73^	1994–1995	USA: New Mexico	Mixed	66,1	244	526	P C M
Strom^74^	1998–2005	USA: Texas	Hispanic	62,2	176	174	P C M
Suzuki^75^	1988–2004	Japan	Japanese	NA	257 in total		H C M
Whittemore^76^	1987–1991	USA, Canada	Mixed	NA, mean age at interview: 71	1500	1581	P C M
Zhu^77^	1989–1991	USA: Washington State	Caucasian	64	175	258	P D M

A P–population based controls, H-hospital based controls, C–cumulative sampling, D–density sampling, M–age-matched controls.

B The year of publishing. The period of collecting the data not reported.

C Calculated from the reported distribution of age of the cases at diagnosis.

NA–not available.

**Table 2:** Summary of the cohort-based studies included in the analysis.

Author	Date	Place	Race	Mean age at diagnosis	Design	Cohort
Brandt^6^	1961–2006	Sweden	Caucasian	NA, aged younger than 75	Cohort study	3,900,000 men from the Swedish cancer registry
Cerhan^78^	1987–1995	USA, Iowa	Caucasian	73,6 A	Prospective cohort study	1557 population-based controls from case-control study in Iowa from 1987–1989
Chen^49^	1990–2004	USA	Caucasian	NA	Prospective cohort study B	43494 men from the Health Professionals Follow-Up Study cohort
Kalish^79^	1987–1997	USA, Boston area	Caucasian	65,2 A	Retrospective cohort study	1149 men from the Massachusetts Male Aging Cohort
Kerber^7^	1966–2000	USA, Utah	Caucasian	NA	Nested case-control, density samping	11572 cases and 11572 controlsfrom the Utah Population Database
Park^80^	1993–2006	USA, California	Mixed	NA	Nested case-control, cumulative sampling	729 cases and 729 controls from the Multiethnic Cohort
Schuurman^81^	1986–1992	The Netherlands	Caucasian	64,2 A	Prospective cohort study	52879 men from the Municipal population registries
Sun^82^	1992–2006	USA	Caucasian	NA	Nested case-control, density sampling	1157 cases and 1157 controls from the Prostate Lung and Ovarian Cancer Screening Trial cohort

A Calculated from the reported distribution of age of the cases at diagnosis.

B Data on exposure to family history was available at baseline, however the updated data from 1996 was used in the analysis.

### Data analysis

The combined estimates were expressed with the ratio of incidence rates (RR) among those exposed and those not exposed. The hazard ratios, the odds ratios from the logistic regression models, and the odds ratios calculated from the contingency tables were assumed to estimate the rate ratios. This measure of association was considered appropriate to express the combined estimates for several reasons. Firstly, the hazard ratios, which can be estimated in cohort studies and density sampling case-control studies [83], are valid estimates of the rate ratios [84]. Also the odds ratios from the logistic regression models from density sampling case-control studies can be used to estimate the rate ratios without any adjustments [85]. Moreover, the bias introduced by estimating the rate ratios with the odds ratios is in general smaller than when the risk ratios are estimated from the odds ratios [85].

When only raw data in a case-control study with age-matched controls was available, the odds ratio and the confidence interval were calculated from the contingency table. In case several measures were available, the one adjusted for more variables was preferred. If the results were reported in strata, the fixed effects model was used to obtain the pooled estimate from the study. Similarly to the previous meta-analyses on familial clustering of prostate cancer [3]–[5], the estimates for men with affected father relative to men without affected father were pooled with those based on the reference category of men without family history in first-degree family members. The same analysis strategy was applied for men with affected brother(s). The estimates based on the reference categories ‘men without affected brothers’ and ‘men without affected first-degree relatives’ were pooled in one analysis.

In order to assess the public health implications of our findings, we estimated the attributable fractions among those with a family history of prostate cancer [86]. The measure was defined as the proportion of the disease incidence attributable to the exposure. The attributable fraction among those exposed can be expressed as: AF_E_  =  (*RR*-1)/*RR* [84]. We estimated AF_E_ by plugging in our estimates of the rate ratios in the equation, which is often referred to as the Mantel-Haenszel approach [87]. The confidence bounds were obtained using the Wald method [88], with the standard deviations estimated by the Monte Carlo simulation [89].

The estimates from the individual studies were combined on the log scale. The Cochran's Q statistic showed evidence of heterogeneity greater than expected by the sampling variance alone. Therefore, we used the random effects model to obtain the combined estimates. For every combined estimate the funnel plot, i.e. a plot of effects estimates versus their standard error, was visually examined and the Egger's asymmetry test [90] was used in order to assess the presence of a publication bias. A meta-regression was carried out to investigate the effect of study design (cumulative sampling case-control versus other), ethnicity (Caucasian versus other), country (US versus other) and publication year on the rate ratio for first-degree family history. The effect of ethnicity on the combined estimate was estimated using the mixed effects model. To evaluate the effect of using the odds ratios from the cumulative-sampling case-control studies as estimates of the rate ratio, we also estimated the rate ratio for first-degree family history using only the cumulative-sampling case-control studies and compared it with the rate ratio based only on the density-sampling case-control studies. Sensitivity of the findings was examined by recalculation of the pooled association sizes after exclusion of studies one by one. As all studies reporting familial aggregation for men under 65 years old also provided data for the age group 65 and older, the paired t-test was used to assess the significance of the difference. The analysis was conducted in SAS (version 9.2; Cary, NC, USA). The rmeta package of the R software (version 2.11.1) was used for the plots.

## Results

### Pooled estimates

The pooled rate ratio for men with first-degree family history, i.e. affected father or/and brother(s), was based on 19 case-control [30], [57]–[60], [62]–[68], [70]–[72], [74]–[77], 3 nested case-control [7], [80], [82] and 4 cohort studies [6], [49], [78], [81]. 18 independent studies [6], [30], [49], [55]–[57], [60], [61], [63]–[66], [70], [71], [73], [75], [78], [81] provided data for men with history of prostate cancer in father and 16 [6], [30], [49], [57], [61], [63]–[66], [70], [71], [73], [75], [78], [79], [81] for men with history of prostate cancer in a brother(s). The combined estimate for history of prostate cancer in a second-degree relative was based on 5 studies [7], [58], [64], [66], [70]. Seven studies [6], [57], [62], [63], [66], [71], [76] were used to estimate the rate ratio for 2 or more affected first-degree family members. Five studies [6], [49], [63], [65], [71] provided data for the different age groups.

**Figure 1:**
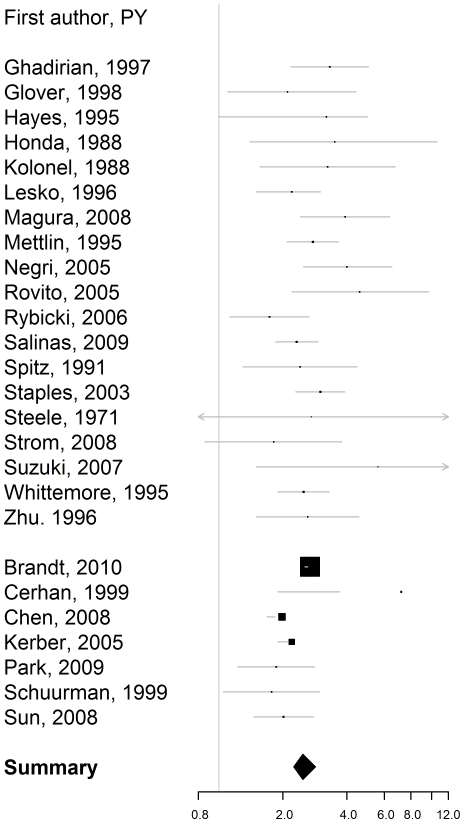
Rate ratio of prostate cancer for first-degree family history, i.e. affected father or brother relative to no first-degree family history. Estimates from the case-control studies are presented at the top. They are separated from the estimates from the cohort-based studies with a line break.

**Figure 2:**
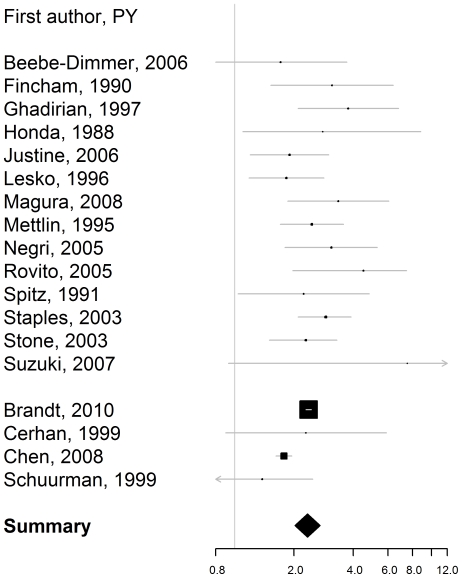
Rate ratio of prostate cancer for a history of prostate cancer in father. Estimates from the case-control studies are presented at the top. They are separated from the estimates from the cohort studies with a line break.

**Figure 3:**
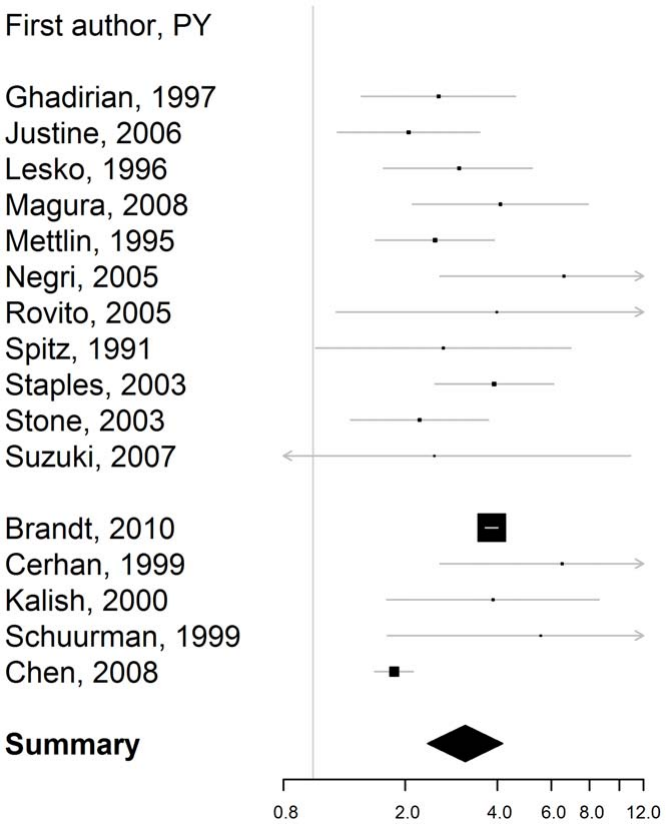
Rate ratio of prostate cancer for a history of prostate cancer in brother(s). Estimates from the case-control studies are presented at the top. They are separated from the estimates from the cohort studies with a line break.

The combined rate ratios and the corresponding attributable fractions among those exposed are reported in Table 3. The rate ratios for firstdegree family history, i.e. affected father or/and brother(s) (Figure 1), affected father (Figure 2), and affected brother(s) (Figure 3) were bigger than 2, with the confidence level 95%. The estimated rate ratio for two or more affected first-degree relatives equaled 4.39 (95% confidence interval: 2.61–7.39). The rate ratio for first-degree family history was significantly higher for men younger than 65, than for men aged 65 or older, p = 0.002.

**Table 3:** Estimates of the rate ratios and the attributable fractions among men with different types of family history.

Type of clustering	RR (95% CI)	AF_E_ (95% CI)	N
1st degree relatives			
For all men	2.48 (2.25–2.74)	59.7% (55.6–63.5%)	26
For men before the age of 65	2.87 (2.21–3.74)	65.2% (57.7–71.4%)	5
For men aged 65 or older	1.92 (1.49–2.47)	47.9% (37.1–56.8%)	5
Affected father	2.35 (2.02–2.72)	57.4% (50.7–63.1%)	18
Affected brother(s)	3.14 (2.37–4.15)	68.1% (58.1–75.7%)	16
2+ 1st degree relatives	4.39 (2.61–7.39)	77.2% (65.4–85.0%)	7
2nd degree relatives	2.52 (0.99–6.46)	60.4% (19.8–80.4%)	5

RR: Rate Ratio, AF_E_: attributable fraction among those exposed, N: number of studies the estimates are based on.

59.7% of the incidence of prostate cancer in men with an affected first-degree relative could be attributed to this risk factor (CI: 55.6–63.5%). When two or more first-degree family members were affected, the attributable fraction equaled 77.2% (CI: 65.4–85.0%). For men younger than 65, the estimated attributable fraction equaled 65.2% (CI: 57.7%–71.4%) and for men 65 or older 47.9% (CI: 37.1–56.8%).

### Publication bias and sensitivity analysis

**Figure 4:**
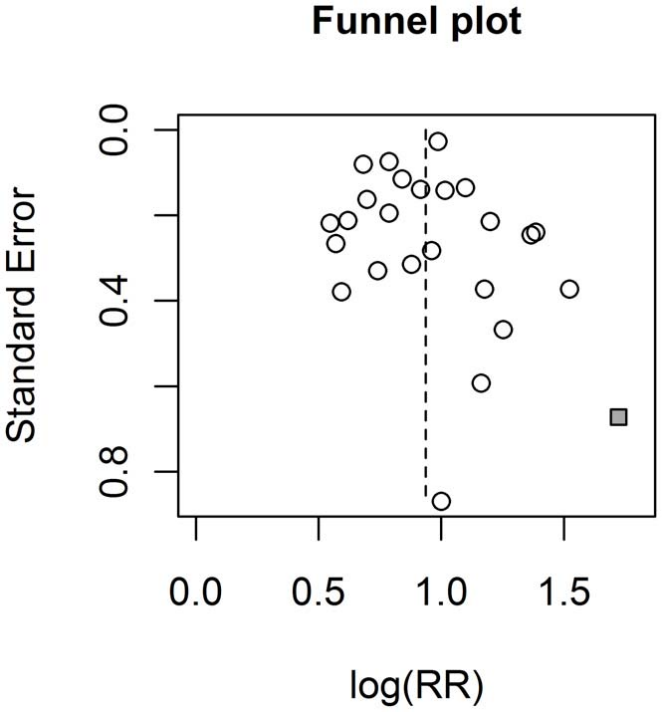
Funnel plot for affected first-degree relatives. The study of Suzuki et al., which may be subject to publication bias, is indicated with a square.

The results of the Egger's test for first-degree family history (p = 0.99), affected father (p = 0.86), affected brother(s) (p = 0.33), and affected second-degree relative (p = 0.06) did not indicate a publication bias. The funnel plot for first-degree family history suggests a potential publication bias (see Figure 4). In particular, the rate ratio for the study by Suzuki et al. [75], a relatively small study, is by far the largest from all the studies included in the analysis. The sensitivity analysis showed that neither a decision to include this study, nor any other shifted the estimate of the rate ratio for first-degree family history or affected father more than by 0.1. Analysis conducted without the study by Chen et al. [49] gave the rate ratio for affected brother(s) 0.18 bigger than the estimate based on all the studies. Excluding none of the other studies led to a change by more than 0.12.

The combined estimate for men aged 65 or older did not change by more than 0.1, when one of the studies it was based on was excluded. For the age category of men younger than 65, it increased from 2.87 to 3.38 when the study by Chen et al. [49] was not included, which was the largest change of the estimate when one of the studies it was based on was excluded. The rest of the estimates appeared to be more sensitive to changes in the sample of studies. The studies by Magura et al. [64] and Kerber et al. [7] had the largest effect on the estimate of the rate ratio for affected second-degree family members. Excluding them changed the estimate from 2.52 to 2.08 and 3.29 respectively. Not including the study by Staples et al. [71] in the sample used to estimate the rate ratio for two or more affected first-degree relatives led to a decrease of the estimate from 4.39 to 3.89, and not using the study by Lesko et al. [63] to an increase to 4.96, which were the largest changes of the estimate when one of the studies it was based on was excluded. Including the study by McCahy et al. [22] would not change any of the combined estimates by more than 0.1.

The meta-regressions showed that neither the study design, country, nor year of publication had a significant influence on the combined estimates for first-degree relatives (p = 0.40 for study design, p = 0.08 for country, and p = 0.29 for publication year). The estimated multiplicative effect of ethnicity on the rate ratio equaled 1.04 (CI:0.83–1.30). The estimated rate ratio equaled 2.50 for Caucasian populations and 2.41 for other populations. This difference was not significant, p = .75. The estimated rate ratio based only on the cumulative-sampling case-control studies and only on the density-sampling case control studies equaled 2.61 (CI:2.25–3.02) and 2.44 (CI:2.08–2.87) respectively.

## Discussion

We identified 74 articles reporting information on the association between family history and prostate cancer from studies conducted in 16 countries in North America, Europe, Asia and Australia. The identified studies differed regarding the study design, the analysis, the way family history was ascertained, the investigated type of clustering, and the reference category that they used. 25 case-control studies and 8 cohort-based studies with non-overlapping populations from 8 countries and 4 continents met the inclusion criteria and reported data on the considered types of clustering. According to our estimates, almost 60% of the prostate cancer incidence among men with first-degree family history is attributable to this risk factor.

The sensitivity analysis showed that the individual study results had a small influence on the pooled estimates of the rate ratio for first-degree family history, affected brother(s), and affected father. The results for affected second-degree relatives and to 2 or more affected first-degree family members, which are based on a small number of studies, are more sensitive to changes in the sample of studies used in the analysis and, therefore, should be treated with caution. The meta-regression and the similarity between the combined estimates based only on the cumulative-sampling case-control studies and only on the density-sampling case-control studies suggested that using the odds ratios from the cumulative-sampling case-control studies as estimates of the rate ratios did not substantially bias our estimates.

We did not attempt to identify unpublished studies. However, neither the visual examination nor the statistical procedures suggested that publication bias could have an important effect on the estimates. With the exception of the study by Glover et al. [58], the studies included in the analysis were conducted in developed countries, most of them in the USA. Our analysis did not suggest that a difference in the strength of familial clustering between the USA and other countries exists. However, generalizing the results to males not living in developed countries may be inappropriate. The population in most of the considered studies was Caucasian. However, the meta-regression showed that the effect of ethnicity on the rate ratio for first-degree family history was small and not significant. This finding suggests that the strength of the association between family history and prostate cancer for Caucasian males is similar as in other populations.

The amount of evidence on the relationship between family history and prostate cancer that was available in our study was much larger, than in the previous meta-analysis. Since 2003, the strength of the associations has been investigated in a number of new populations and a more recent data has been reported from the Swedish cancer register and the Utah population database. This allowed using stringent inclusion criteria and at the same time retaining a substantial number of studies. In contrast to the previous works, several measures were taken in order to improve the quality of the analysis. To avoid a possible bias caused by confounders the studies among specific populations such as smokers [10] were excluded. As referring to prostate examination may occur more frequently among men with prostate cancer family history, the cross-sectional data gathered among males referred to examination by a doctor [11], [12], [14] was not used. Homogeneity of the studies was assured by including only the studies in which family history was defined and corresponding to one of the investigated types of clustering. Finally, as pooling of the studies required the assumption of independence, overlaps in study populations were avoided.

The results confirm the conclusions made in the previous meta-analyses [3]–[5] and support the American Cancer Society guidelines. We observed more than a 2-fold increase in the incidence rate of the disease for all of the investigated types of familial clustering, meaning that over 50% of prostate cancer cases among men with a certain type of family history are attributable to familial clustering of the disease. Having a brother with prostate cancer appears to be associated with a larger increase in the incidence rate than being a son of a father with prostate cancer. The incidence rate increases with an increasing number of affected family members. Family history appears to increase the incidence rate of prostate cancer for males younger than 65 more, than for males aged 65 and older, which suggests the relative importance of genetic factors and/or shared environment and/or food factors in an early onset of prostate cancer. In line with our conclusions, the American Cancer Society (ACS) recommends that men at average risk should be offered testing beginning at age 50, and that men at increased risk for prostate cancer, such as those with a history of the disease in a father or brother at a young age, should begin testing with both the prostate specific antigen blood test and the digital rectal examination at age 45, or even younger if they have multiple relatives with the disease.

## Supporting Information

Figure S1 Flow of Included Studies.
(DOC)
