# Supplementary material for: An Epidemiological Reappraisal of the Familial Aggregation of Prostate Cancer: A Meta-Analysis
Source: PLoS One. 2011 Oct 31;6(10):e27130. doi: 10.1371/journal.pone.0027130 (PMC3205054; doi:10.1371/journal.pone.0027130)
Supplement: Figure S1 — Flow of Included Studies. (DOC) [file pone.0027130.s001.doc]

Potentially relevant studies identified via PUBMED (801)

Course of the disease (76)

Attitudes and knowledge about cancer (56)

Case-studies (3)

Methodological (16)

Molecular genetics (132)

Other cancers/diseases (79)

Other not related to familial clustering of the cancer (10)

Other risk factors (251)

Reviews (86)

Specific populations (39)

Retrieved (53)

Cross-sectional studies (7)

Family history not defined (2)

Inadequate control for age (3)

None of the participants reported family history (1)

Other types of familial clustering (5)

Outlying results (1)

Duplication in study population (22)

Identified by browsing references (21)

Studies included in meta-analysis (33)

First degree relative affected (26)

Affected father (18)

Affected brother (16)

Affected second-degree relative (5)

More than one first-degree family member (7)

Disease onset before 65 in first degree relatives (5)

Potentially appropriate studies to be included in the meta-analysis (74)
